# Supplementary material for: High measles and rubella vaccine coverage and seroprevalence among Zambian children participating in a measles and rubella supplementary immunization activity
Source: PLOS Glob Public Health. 2025 Aug 29;5(8):e0003209. doi: 10.1371/journal.pgph.0003209 (PMC12396667; doi:10.1371/journal.pgph.0003209)
Supplement: S1 Table — The analysis was restricted to children 12 months and older. The outcome was no MR doses prior to the SIA. Univariable ORs were adjusted for age in years. Analysis with SIA site type (outreach vs fixed) was restricted to health facilities with both fixed and outreach locations. Bold indicates p < 0.05. (DOCX) [file pgph.0003209.s001.docx]

**S1 Table. Characteristics associated with not receiving measles-rubella vaccine prior to the SIA**

|  | **Odds ratios (95% CI)** | |
| --- | --- | --- |
|  | **Choma District** | **Ndola District** |
| Rural setting (vs. urban) | 0.8 (0.3, 2.3) | 0.4 (0.0, 1.7) |
| Outreach site (vs. fixed) | 1.1 (0.4, 2.6) | 1.4 (0.6, 3.4) |
| Travel time to campaign site |  |  |
| > 30 minutes (vs. < 30) | 1.9 (0.8, 4.5) | 1.5 (0.7, 3.0) |
| Siblings (v. only child) |  |  |
| 1 sibling < 5 | 1.1 (0.4, 2.5) | 1.8 (0.9, 3.5) |
| 2 or more siblings < 5 | 0.0 (0.0, >99.99) | 0.0 (0.0, >99.99) |
| Did not receive BCG | 1.9 (0.1, 9.7) | 2.3 (0.4, 8.0) |
| Did not receive DTP | 4.1 (0.2, 23.4) | **7.9 (1.7, 26.9)** |
| Maternal education primary or less (v. secondary or higher) | 0.9 (0.4, 1.9) | 1.1 (0.5, 2.0) |

The analysis was restricted to children 12 months and older. The outcome was no MR doses prior to the SIA. Univariable ORs were adjusted for age in years. Analysis with SIA site type (outreach vs fixed) was restricted to health facilities with both fixed and outreach locations. Bold indicates p < 0.05.
