# Supplementary material for: High measles and rubella vaccine coverage and seroprevalence among Zambian children participating in a measles and rubella supplementary immunization activity
Source: PLOS Glob Public Health. 2025 Aug 29;5(8):e0003209. doi: 10.1371/journal.pgph.0003209 (PMC12396667; doi:10.1371/journal.pgph.0003209)
Supplement: S2 Table — This analysis was restricted to children 24 months and older with at least 1 MR dose prior to the SIA. The outcome was no receipt of the second MR dose prior to the SIA. Univariable ORs adjusted for age in years. Analysis with SIA site type (outreach vs fixed) was restricted to health facilities with both fixed and outreach locations. Bold indicates p < 0.05. (DOCX) [file pgph.0003209.s002.docx]

**S2 Table. Characteristics associated with missing the second MR dose prior to SIA among those with at least 1 dose.**

|  | **Odds ratios (95% CI)** | |
| --- | --- | --- |
|  | **Choma District** | **Ndola District** |
| Rural setting (vs. urban) | 1.7 (0.8, 3.9) | 0.9 (0.3, 2.1) |
| Outreach site (vs. fixed) | 0.8 (0.5, 1.3) | **1.8 (1.0, 3.3)** |
| Travel time to campaign site |  |  |
| > 30 minutes (vs. < 30) | 0.7 (0.5, 1.1) | 1.1 (0.6, 1.8) |
| Siblings (v. only child) |  |  |
| 1 sibling < 5 | 1.3 (0.8, 2.0) | 1.5 (0.9, 2.5) |
| 2 or more siblings < 5 | **3.4 (1.1, 9.1)** | **4.0 (1.4, 10.1)** |
| Did not receive BCG | 0.9 (0.1, 3.4) | 0.4 (0.0, 2.2) |
| Did not receive DTP | >99.99 | 1.2 (0.1, 7.8) |
| Maternal education primary or less (v. secondary or higher) | **1.9 (1.2, 3.0)** | 1.2 (0.7, 1.9) |

This analysis was restricted to children 24 months and older with at least 1 MR dose prior to the SIA. The outcome was no receipt of the second MR dose prior to the SIA. Univariable ORs adjusted for age in years. Analysis with SIA site type (outreach vs fixed) was restricted to health facilities with both fixed and outreach locations. Bold indicates p < 0.05.
