# Supplementary material for: High measles and rubella vaccine coverage and seroprevalence among Zambian children participating in a measles and rubella supplementary immunization activity
Source: PLOS Glob Public Health. 2025 Aug 29;5(8):e0003209. doi: 10.1371/journal.pgph.0003209 (PMC12396667; doi:10.1371/journal.pgph.0003209)
Supplement: S3 Table — (DOCX) [file pgph.0003209.s003.docx]

**S3 Table. Characteristics associated with measles seronegativity**

|  | **Choma District** | **Ndola District** |
| --- | --- | --- |
| Rural setting (vs. urban) | 1.0 (0.6, 1.6) | 0.8 (0.3, 1.5) |
| Outreach site (vs. fixed) | 0.9 (0.6, 1.3) | 0.8 (0.5, 1.3) |
| Travel time to campaign site |  |  |
| > 30 minutes (vs. < 30) | 1.0 (0.7, 1.4) | **0.6 (0.4, 0.9)** |
| Siblings (v. only child) |  |  |
| 1 sibling < 5 | **1.4 (1.0, 2.0)** | 1.0 (0.6, 1.5) |
| 2 or more siblings < 5 | **3.2 (1.4, 6.9)** | 0.2 (0.01, 0.9) |
| Did not receive BCG | **2.3 (1.0, 4.7)** | **6.0 (2.8, 12.7)** |
| Did not receive DTP | 2.4 (0.7, 6.8) | 2.4 (0.7, 7.0) |
| Maternal education primary or less (v. secondary or higher) | 1.2 (0.8, 1.6) | 1.1 (0.8, 1.6) |

The outcome was measles seronegativity. ORs were adjusted for age in years. Analysis of SIA site type (outreach vs fixed) was restricted to health facilities with both fixed and outreach locations. Bold indicates p < 0.05.
