# Supplementary material for: High measles and rubella vaccine coverage and seroprevalence among Zambian children participating in a measles and rubella supplementary immunization activity
Source: PLOS Glob Public Health. 2025 Aug 29;5(8):e0003209. doi: 10.1371/journal.pgph.0003209 (PMC12396667; doi:10.1371/journal.pgph.0003209)
Supplement: S1 Fig — Children were excluded from this analysis if vaccine receipt was based on recall, if the dosing date was prior to their date of birth, or if the MR2 receipt date was on or prior to the date of MR1 receipt. Remaining children with a card but no documented evidence of MR were right-censored at 59 months. (DOCX) [file pgph.0003209.s004.docx]

**
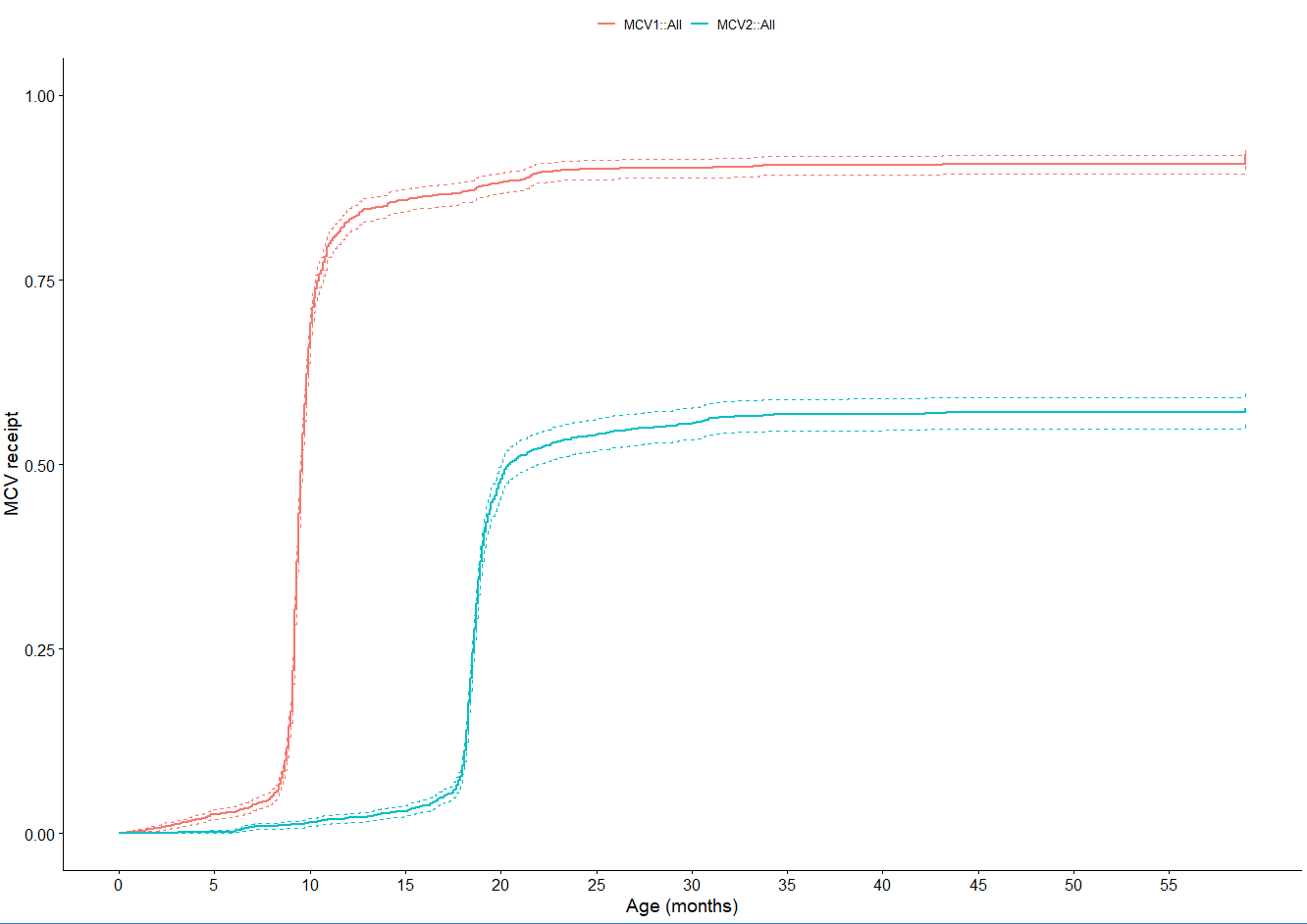
**

**S1 Fig. Age at routine MR receipt.** Children were excluded from this analysis if vaccine receipt was based on recall, if the dosing date was prior to their date of birth, or if the MR2 receipt date was on or prior to the date of MR1 receipt. Remaining children with a card but no documented evidence of MR were right-censored at 59 months.
