# Supplementary material for: High measles and rubella vaccine coverage and seroprevalence among Zambian children participating in a measles and rubella supplementary immunization activity
Source: PLOS Glob Public Health. 2025 Aug 29;5(8):e0003209. doi: 10.1371/journal.pgph.0003209 (PMC12396667; doi:10.1371/journal.pgph.0003209)
Supplement: S2 Fig — Dashed vertical lines represent anticipated age of MR1 and MR2 receipt (MR1, 9 months and MR2, 18 months). (DOCX) [file pgph.0003209.s005.docx]

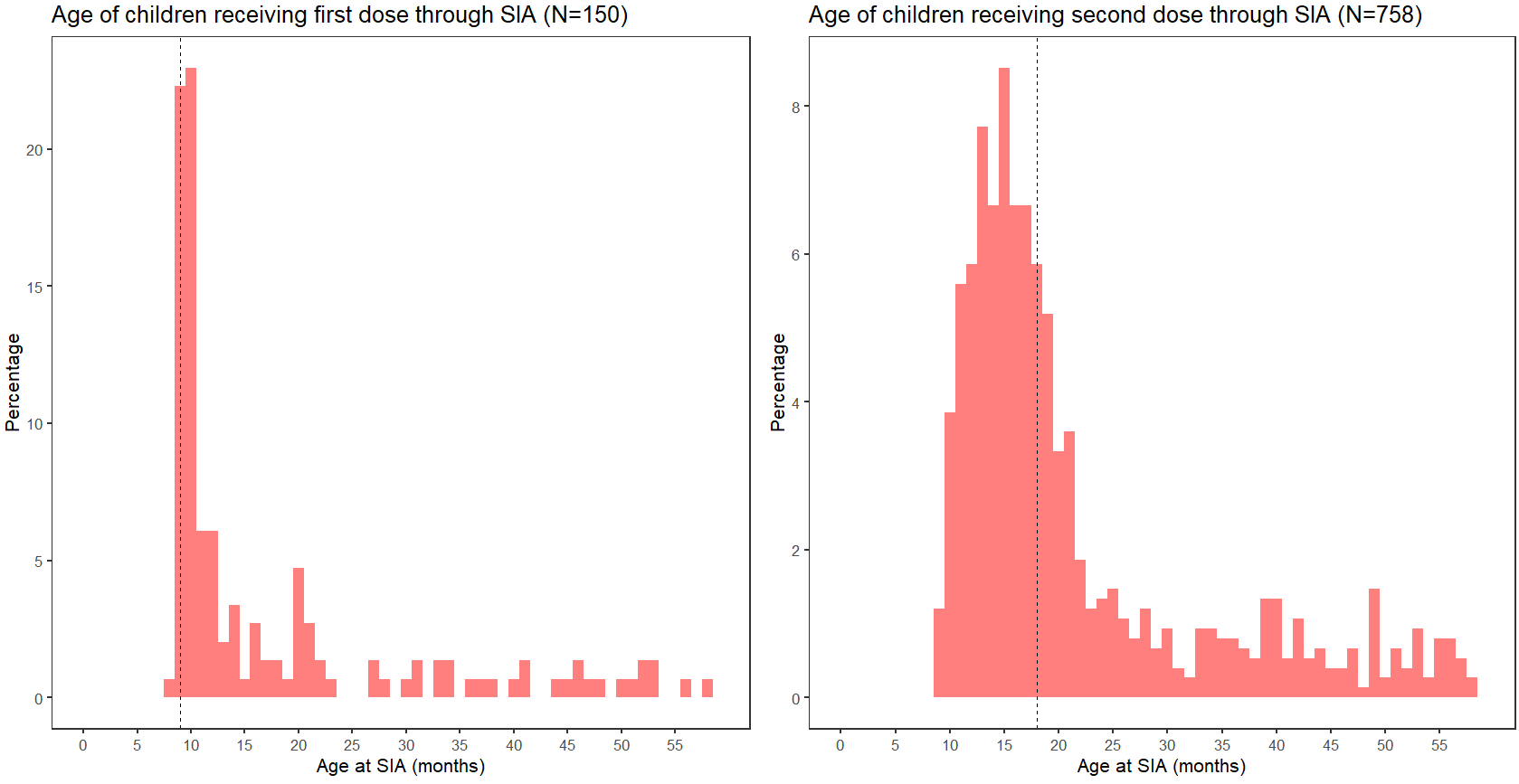


**S2 Fig. Age at vaccination during the SIA for children receiving their first or second measles vaccine dose through the SIA.** Dashed vertical lines represent anticipated age of MR1 and MR2 receipt (MR1, 9 months and MR2, 18 months)
