# Supplementary figures and images for: High measles and rubella vaccine coverage and seroprevalence among Zambian children participating in a measles and rubella supplementary immunization activity
Source: PLOS Glob Public Health. 2025 Aug 29;5(8):e0003209. doi: 10.1371/journal.pgph.0003209 (PMC12396667; doi:10.1371/journal.pgph.0003209)

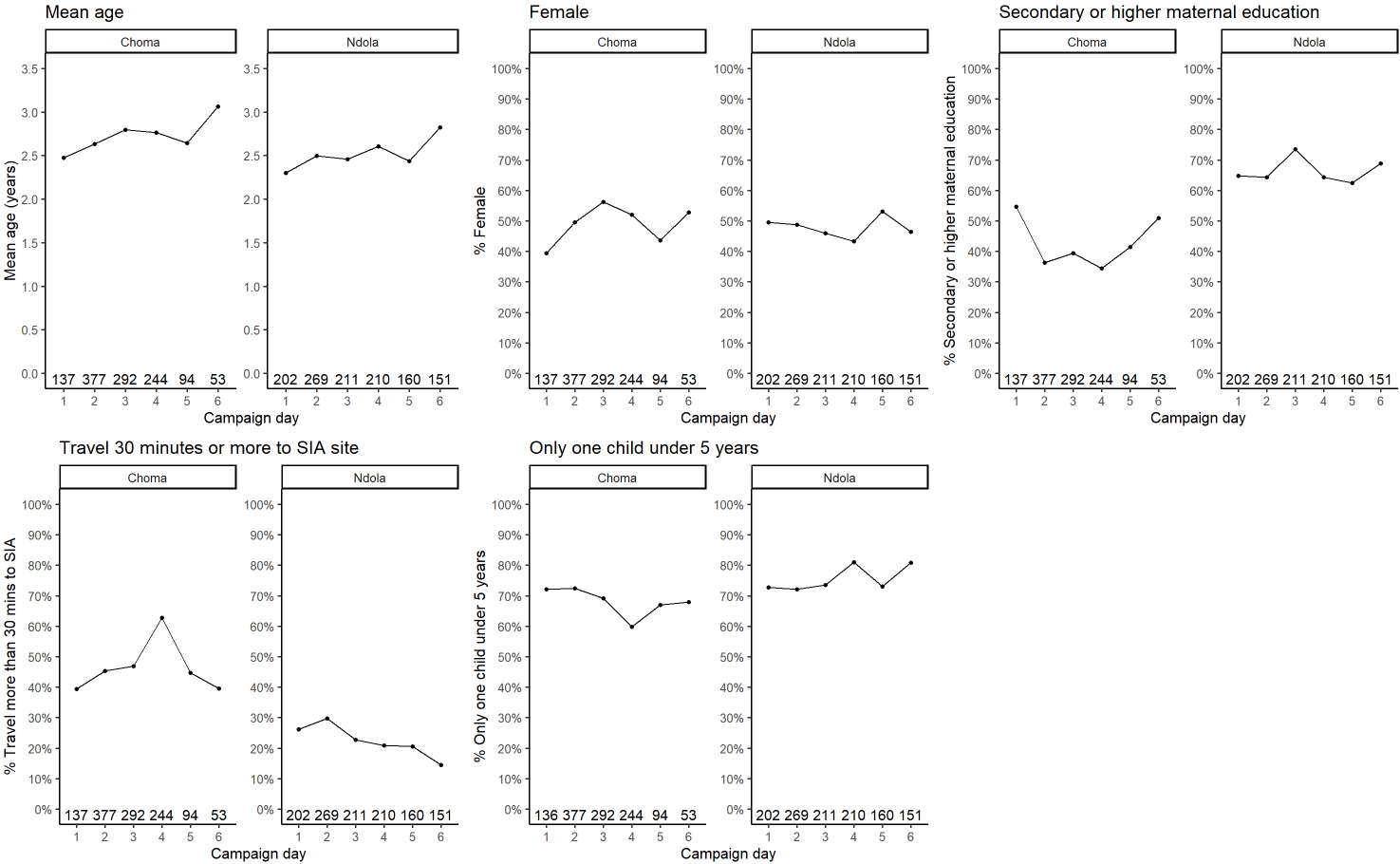


**S3 Fig. Variability in descriptive characteristics of children by SIA day.**

Supplement: S3 Fig — (DOCX) [file pgph.0003209.s006.docx]

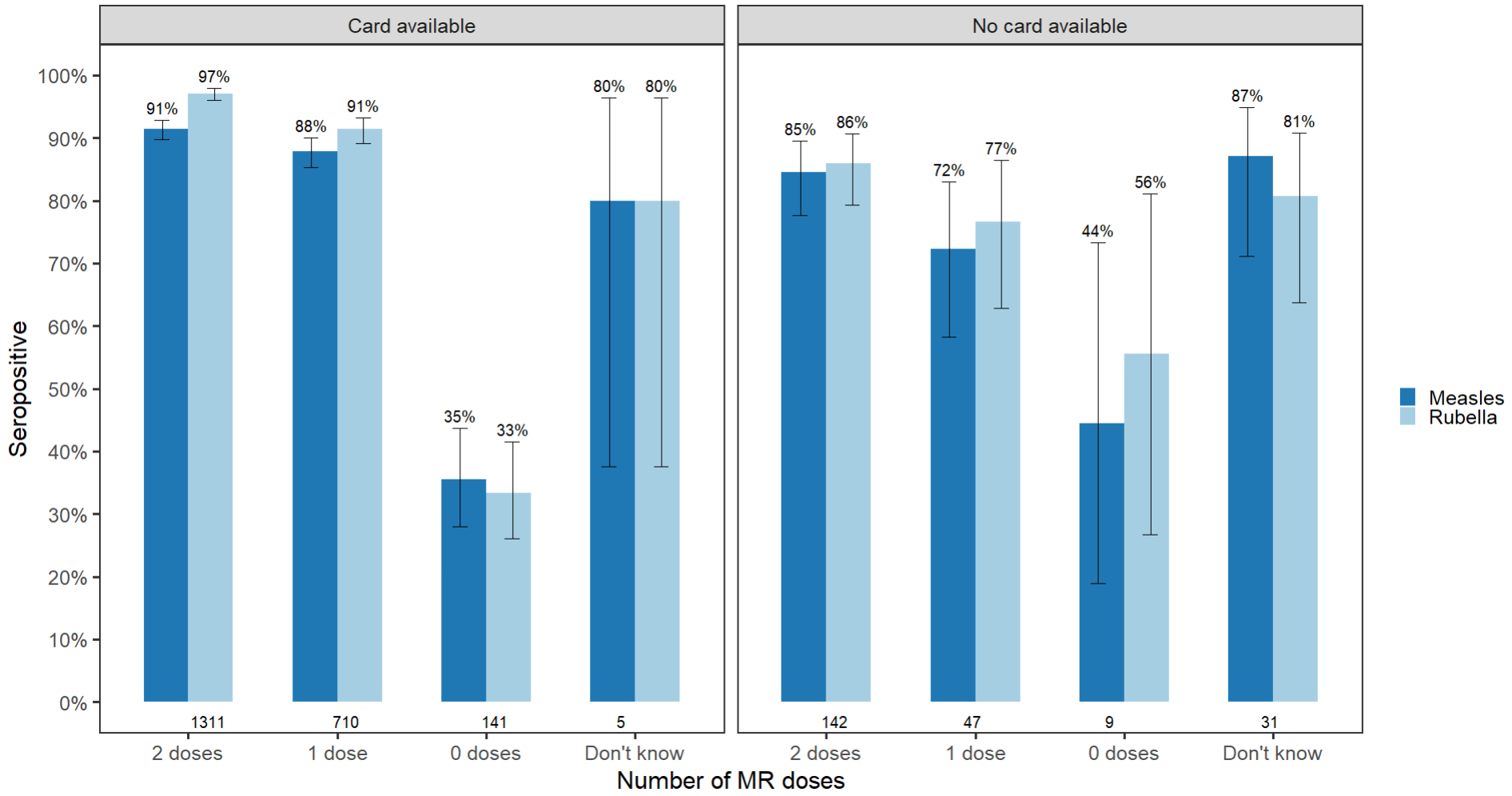


**S5 Fig. Measle and rubella seropositivity by number of doses and card availability.**

Supplement: S5 Fig — (DOCX) [file pgph.0003209.s008.docx]
