# Supplementary material for: High measles and rubella vaccine coverage and seroprevalence among Zambian children participating in a measles and rubella supplementary immunization activity
Source: PLOS Glob Public Health. 2025 Aug 29;5(8):e0003209. doi: 10.1371/journal.pgph.0003209 (PMC12396667; doi:10.1371/journal.pgph.0003209)
Supplement: S4 Fig — Both districts combined. Restricted to health facilities with both fixed and outreach locations and excluding children < 12 months. P-value for variability by day in percent zero-dose from logistic regression model adjusted for age and district: fixed site, 0.60; outreach site, 0.07. (DOCX) [file pgph.0003209.s007.docx]

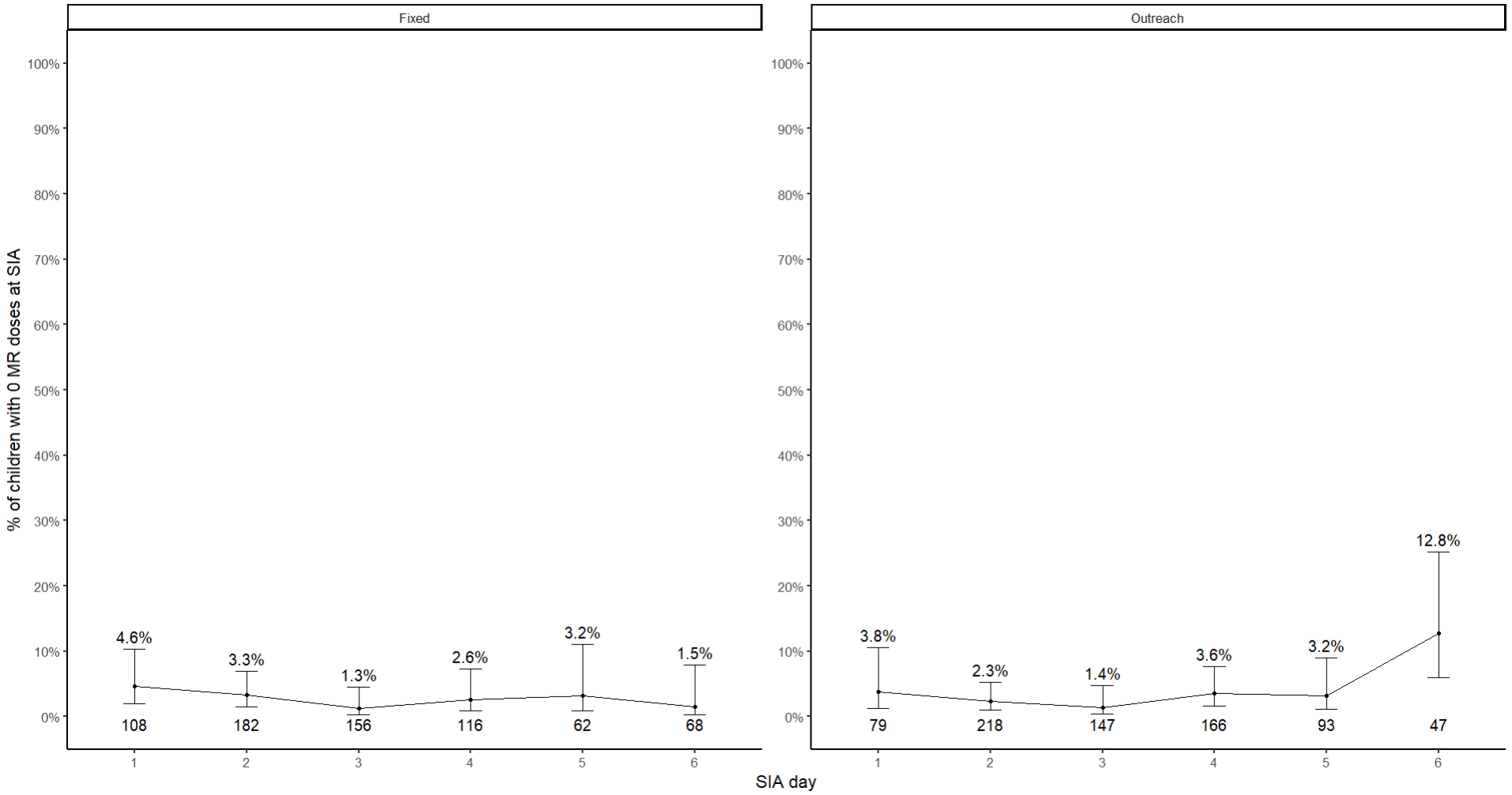


**S4 Fig. Variability by day in the percentage of children with no MR doses prior to the SIA by type of SIA site.** Both districts combined. Restricted to health facilities with both fixed and outreach locations and excluding children < 12 months. P-value for variability by day in percent zero-dose from logistic regression model adjusted for age and district: fixed site, 0.60; outreach site, 0.07.
