# Supplementary material for: High measles and rubella vaccine coverage and seroprevalence among Zambian children participating in a measles and rubella supplementary immunization activity
Source: PLOS Glob Public Health. 2025 Aug 29;5(8):e0003209. doi: 10.1371/journal.pgph.0003209 (PMC12396667; doi:10.1371/journal.pgph.0003209)
Supplement: S6 Fig — Analysis with SIA site type (outreach vs fixed) restricted to health facilities with both fixed and outreach locations. (DOCX) [file pgph.0003209.s009.docx]

**
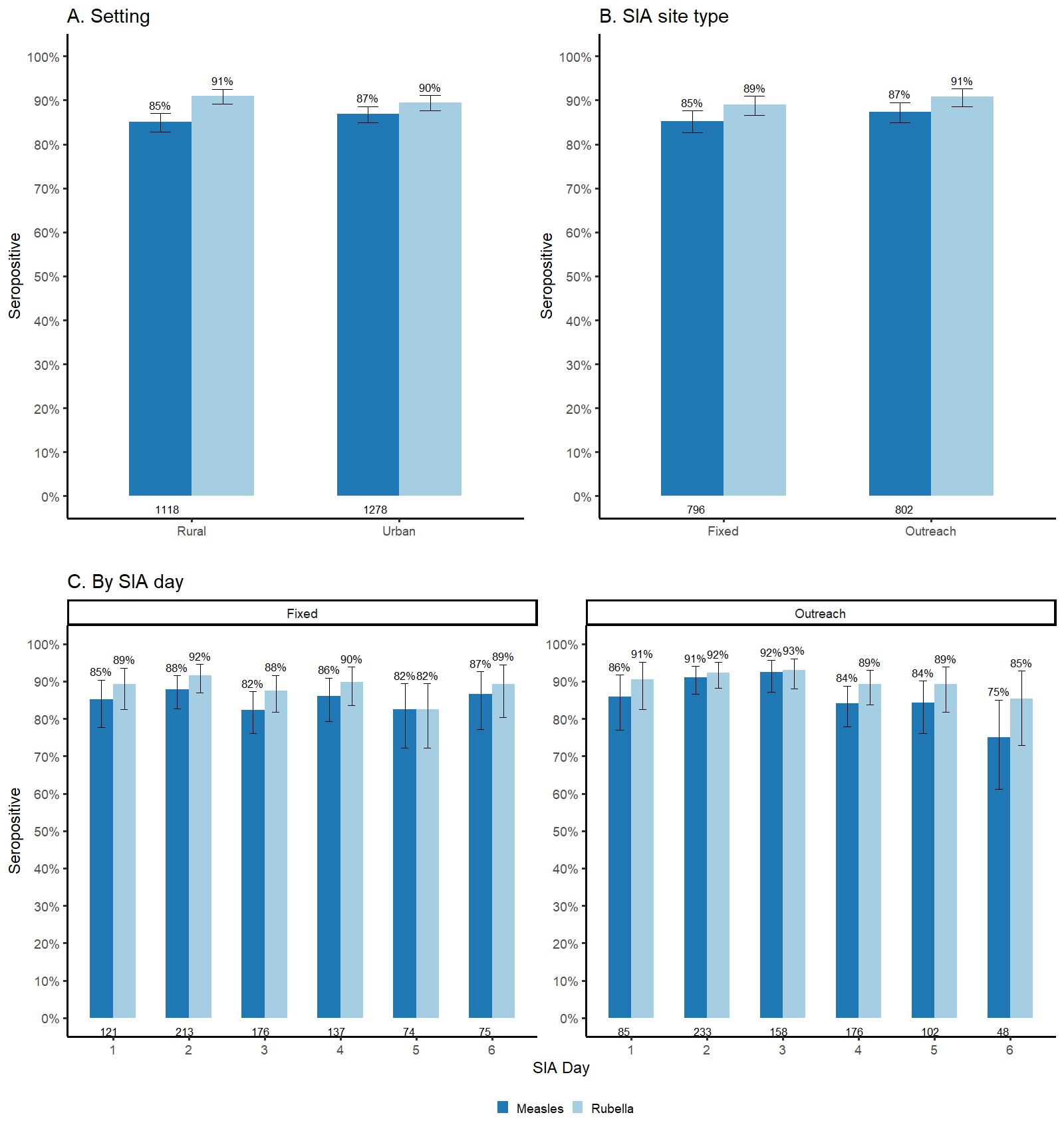
**

**S6 Fig. Measles and rubella seropositivity by setting, SIA type, and campaign day.** Analysis with SIA site type (outreach vs fixed) restricted to health facilities with both fixed and outreach locations.
