# Supplementary material for: High measles and rubella vaccine coverage and seroprevalence among Zambian children participating in a measles and rubella supplementary immunization activity
Source: PLOS Glob Public Health. 2025 Aug 29;5(8):e0003209. doi: 10.1371/journal.pgph.0003209 (PMC12396667; doi:10.1371/journal.pgph.0003209)
Supplement: S1 Text — (DOCX) [file pgph.0003209.s010.docx]

**S1 Text: Supplemental Methods**

*Logistic regression analysis*

We conducted district-specific logistic regression models with MCV receipt (1 or 2 doses) as the binary outcome and campaign site as the predictor, adjusted for the child’s age. The model for MCV2 was restricted to children > 2 years of age. Using the output from these models we estimated the predicted probability of a child 12 months of age having received MR1 or a child 24 months of age having received MR2 at each campaign site.

$MCV1 receipt \sim SIA site + child’s age$

$MCV2 receipt \sim SIA site + child’s age [restricted to children > 2 years]$

We conducted district-specific logistic regression models with MCV receipt (1 or 2 doses) or seropositivity as the binary outcome and the child-level characteristic as the primary predictor (urban/rural, fixed/outreach vaccination site, travel time to the campaign, vaccination card availability, number of siblings below 5y, or missing DTP1), adjusted for the child’s age. The model for MCV2 receipt was restricted to children > 2 years of age.

*Hazard of vaccination analysis*

To estimate the value of the SIA to reach children who may not have otherwise been vaccinated through routine immunization programs, we conducted a “hazard of vaccination”, or *HoV*, analysis. To do this analysis, we estimated the cumulative probability of MR1 and MR2 by month of age for each district using the 2018 Demographic and Health Survey data (Fig 1 in S1 Text). These probabilities are estimated relying on the following variables from children aged 0–36 months: age at the time of survey, whether the child had ever received an MR1 and MR2 vaccine dose (based on vaccination card or report of the parent or guardian), and age at the time of vaccination (if a vaccination card was available). Data were available for 5,670 children in 112 of the 116 districts (e.g., Ndola District N=144, Choma District N=132). To estimate vaccination timing and coverage, we used a modified survival analysis framework that incorporated different types of censoring: exact vaccination dates, caregiver-reported vaccination with missing dates, and unvaccinated children at the time of survey. This approach and estimates, presented in detail by Winter et al. 2023 [1], models the likelihood of vaccination by a given age using two key parameters per district: the lifetime probability of being vaccinated and the rate at which vaccination occurs. To account for spatial correlation across districts and extend the DHS’s provincial-level granularity, we employed a conditional autoregressive (CAR) spatial model. Thus, district-level estimates were informed not only by survey data but also by assumptions embedded in the spatial modeling structure. From these estimates, we estimated the probability that an individual would eventually be vaccinated via routine systems given their age, or their “hazard of vaccination”, as the final probability of vaccination with each dose minus the cumulative probability up to that age.

$$HoV= \int_{0}^{A} v(t)dt-\int_{0}^{a} v(t)dt$$

Where $A$ is the maximum age of vaccine receipt and $a$ is the age of interest.

To estimate the population *HoV,* each child captured within the SIA yet to receive a vaccine is assigned the hazard of vaccination for their age as their probability of eventually being vaccinated; children already vaccinated are assigned Pr=1. Probabilities are aggregated to estimate the eventual dose coverage in absence of the SIA for the population receiving the SIA dose.


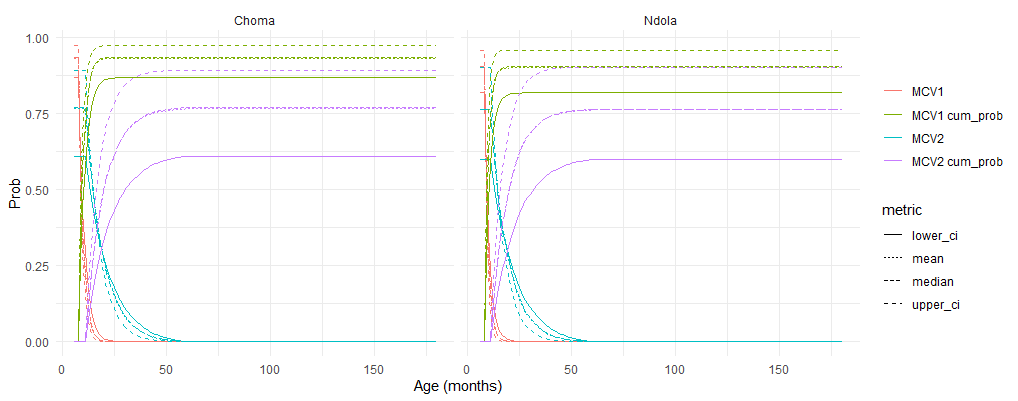


**Fig 1. Incident and cumulative probability of vaccination by age, Choma and Ndola, Zambia.**

We can also use the *HoV* to estimate the *vaccine activity efficiency*, or *VAEC*, as

$$VAEC=1-mean\left( HoV \right)$$

and the per dose efficiency of the activity as

$$doses needed to reach 1 never vaccinated child=\frac{1}{VAEC}$$

These three simple metrics enable us to understand just how useful the activity was to reach children who would otherwise not have received the dose.

The For example, we estimate that in Choma, Zambia children have a probability of receiving MR1 by 10 months of age of 0.57 (0.46-0.68), and by 18 months of 0.92 (0.85-0.97). Thus, if a 10-month-old captured by the SIA is unvaccinated, they have a probability that they may have received MR1 of 0.92-0.57 = 0.35. By ignoring this remaining probability that they could still get vaccinated and just assuming because they are currently unvaccinated at the SIA, we are incorrectly assigning more value to the SIA to reach this child than we should, given they still had a 35% chance of getting a routine dose without it. If somehow our entire SIA population was children aged 10 months who had not received a vaccine yet, initial estimates of the value of the SIA would be high, with every child reached currently being unvaccinated. However, using the *HoV* estimates for this population, we can calculate the *vaccine activity efficiency*, or *VAEC*, as

$$VAEC=1-mean\left( HoV \right)$$

and in this example, we find VAEC = 0.65, meaning this activity was 65% efficient at reaching children who would not have otherwise been vaccinated (i.e., 65% of children). We can also convert this to a per dose efficiency as

$$doses needed to reach 1 never vaccinated child=\frac{1}{VAEC}$$

$$=\frac{1}{0.65}= 1.54$$

Thus, it required 1.54 doses to reach 1 child who would never have been vaccinated (“never vaccinated”) in this hypothetical population. The higher this number, the more inefficient an activity.

However, if this SIA vaccinated all 10-month-olds, and 90% of them were already vaccinated, this activity would have a mean *HoV* of 0.935 and *VAEC* = 0.065, meaning it took 15.4 doses to reach 1 child that needed it.

*Quality control and quality assurance*

Four systematically selected specimens per plate were re-tested on the same plate (intraplate variation) and four re-tested on a subsequent plate (interplate variation). In addition to the kit-provided calibrators and controls, two study-specific internal controls were run on each plate. Internal controls were well-characterized specimens obtained from the blood bank at Ndola Teaching Hospital and included a high-positive and low-positive measles control.

The results were monitored in near-real-time by reviewing the calibrators, the kit-provided and study-specific internal controls, and interplate and intraplate specimen repeats. Any issues with plate validity or concerns about the run were flagged for the testing team and the plate was repeated if necessary. For intraplate and interplate repeats, a scatterplot of repeat quantitative values was generated and the R-squared value was calculated (Table 1 in S1 Text). For specimens retested for quality control purposes, the original result was used as final unless there was a qualitative difference, in which case the specimen was re-tested and the most commonly observed qualitative result was selected as the final result.

**Table 1. Correlation coefficients for intraplate and interplate repeats**

|  | Measles | | Rubella | |
| --- | --- | --- | --- | --- |
|  | Intraplate | Interplate | Intraplate | Interplate |
| MRT | 0.8672 | 0.7357 | 0.7750 | 0.8490 |
| TDRC | 0.9864 | 0.7758 | 0.9591 | 0.7941 |

Adjusted R^2^ values after excluding results below the lower limit of detection (measles: 8 mIU/ml, rubella: 0.3 IU/ml) or above upper limit of detection (measles: 5000 mIU/ml; rubella: 200 IU/ml).

*Testing procedures*

There was one technician at MRT and two technicians at TDRC running the assays. Testing was performed by cluster. Measles and rubella testing was done simultaneously from the same elution. All results were entered into a study- and antigen-specific Excel template and uploaded to a central repository once each plate was completed.

For measles IgG antibodies, values >153 mIU/mL after applying the adjustment based on the MBA results were assigned as positive. For rubella IgG antibodies, values >11 IU/mL were assigned as positive, those with values of >8 and <11 were classified as equivocal and those < 8 were classified as negative. Equivocal specimens were treated as positive in analyses.

**Measles IgG Quantitative Adjustment**

*Rationale for quantitative adjustment:* Preliminary results from this study yielded much lower than anticipated estimates of measles IgG seropositivity (62%), given estimated vaccine coverage and results from prior studies conducted in this setting [2, 3]. In addition, prior issues identified by the research team with the Euroimmun measles enzyme immunoassay (EIA) (Euroimmun AG, Lübeck, Germany) after a change was made to one of the calibrators that primarily impacted specimens near the equivocal threshold added to concerns about the low sensitivity of this assay [4]. To evaluate the performance of the assay, a comparative study was conducted.

*Sample selection for the comparative study:* To assess the sensitivity of the EIA, a subsample of 300 DBS specimens were selected from those collected in Ndola District during the serosurvey. The specimens were purposefully selected across the range of values, with more samples selected with values around the thresholds. Half of the specimens had EIA antibody values between 100-200 mIU/mL, 100 had values <100 mIU/mL, and 50 had values >200 mIU/mL. Samples were shipped to the Centers for Disease Control and Prevention (CDC), Viral Preventive Diseases Branch in Atlanta, Georgia, USA for testing using a multiplex bead assay (MBA).

*Measles MBA testing*: The MBA is a serological assay that has been increasingly utilized for its ability to provide high-quality seroprevalence data and to test antigens from multiple pathogens at once, among other factors [5]. The MBA uses a commercially produced whole-virus lysate antigen prepared using MagPlex or MicroPlex beads. In a validation study comparing the MBA to the measles plaque reduction neutralization (PRN) assay, the current “gold standard”, there was a strong correlation between IgG concentrations from the MBA and PRN titers [5]. Compared to PRN, the MBA had a sensitivity of 98% and a specificity of 83%, when a seroprotection cutoff at 153 mIU/mL was used. In the comparative study, samples were tested using a MagPix instrument (Luminex Corp., Austin, Texas), and including background beads with uninfected Vero lysate to account for background signals in the specimens. All plates were run with a standard curve, which was used to calculate observed concentrations.

*Statistical analysis:* For the samples tested using both the EIA and MBA, results from both assays were compared (Fig 2-5 in S1 Text). There appeared to be a linear relationship between the two assay outputs, with the MBA producing consistently higher results than the EIA (Fig 2-3 in S1 Text). As this supported a low sensitivity of the Euroimmun assay, an analysis was performed using linear regression to estimate a correction factor to be applied to the EIA IgG antibody concentrations. We fit models to a subset of data between 8 mIU/mL EIA (the lower limit of detection of the EIA) and 3000 mIU/mL EIA (removing the outlier) (Fig 2 in S1 Text). We employed the 'brms' package, which is a Bayesian statistical package in R, to fit two linear models to the data. We found the log-log linear model fit the data best compared to linear model (Fig 4 in S1 Text). Fig 5 in S1 Text displays a graphical representation of the log-log model fit to the data. The mean estimate of the intercept was 0.495, the mean estimate of the slope was 1.092. The following equation was used to calculate the adjusted mIU/mL to the entire range of EIA values from this study: Adjusted_mIUmL = exp(0.495 + (1.092*ln(EIA_mIUmL)). Adjusted values at or above 153 mlU/mL were considered seropositive. After applying the correction factor to the EIA values, the proportion seropositive increased from 62% to 86% (Fig 6 in S1 Text).


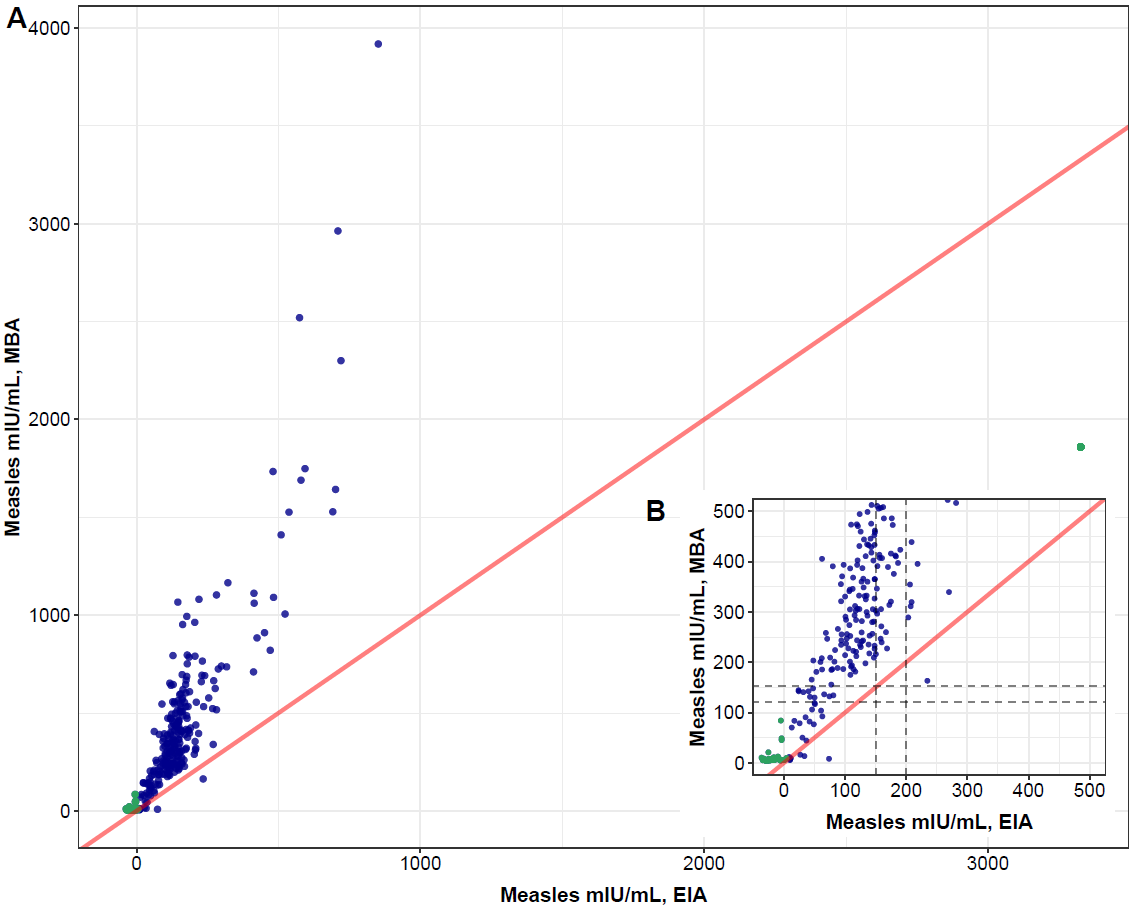


**Fig 2. Scatter plot comparing IgG antibody concentrations from the multiplex bead assay (MBA, y-axis) and enzyme immunoassay (EIA, x-axis) (N=290).** A) Scatter plot of the full range of data and B) scatter plot of data less than 500 mIU/mL. The red line represents perfect agreement between the two tests. The green points represent specimens that per the EIA are >3000 mIU/mL or <8 mIU/mL, which were removed prior to fitting the linear models. The dashed line on the y-axis indicates the MBA cutoff for seroprotection of 153 mlU/mL and 120 mIU/mL. The dashed lines on the x-axis indicate the thresholds for borderline (150 mIU/mL) and positive (200 mlU/mL) in assessing immune status using the EIA.


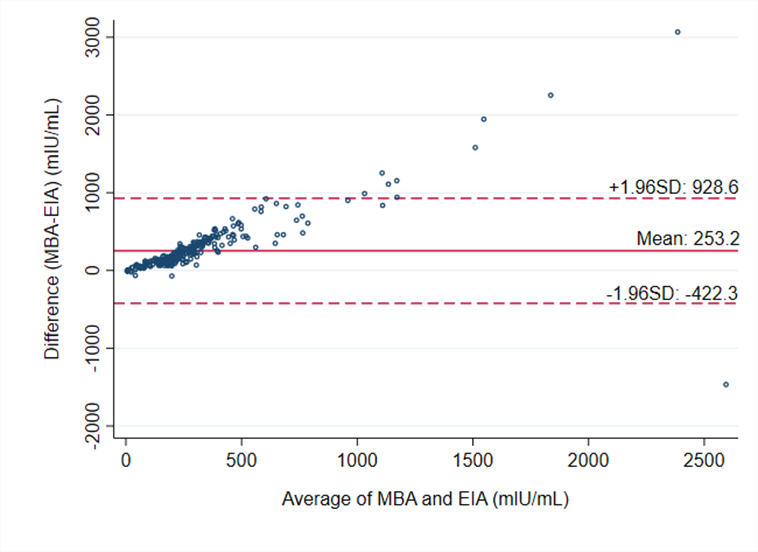


**Fig 3. Bland-Altman plot comparing the difference in IgG antibody concentrations from the multiplex bead assay (MBA) and enzyme immunoassay (EIA), relative to their average antibody concentration.** The solid red line indicates the average difference between MBA and EIA IgG antibody concentrations, while the dashed red lines indicate thresholds for differences between MBA and EIA concentrations that are ± 1.96 standard deviations (SD) from the mean.


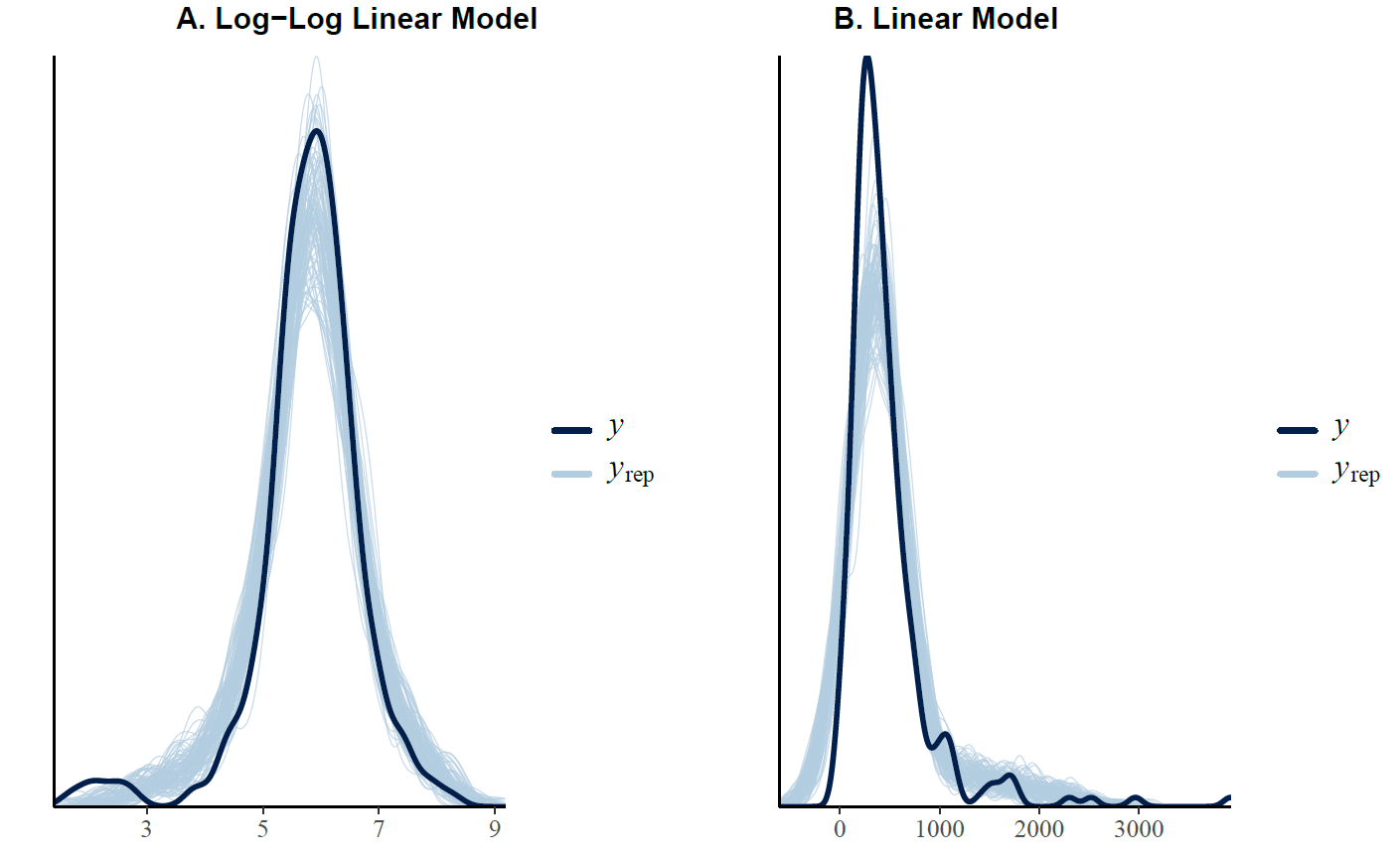


**Fig 4. Distribution of observed and replicated measles IgG antibody concentrations (mIU/mL).** Posterior predictive checks of Log-Log Linear Model (A) and Linear Model (B). The dark line (y) represents the observed MBA data, and the light blue lines (y_rep_) represent predictions of MBA from the posterior distributions. We see that the log-log model fit the data better.


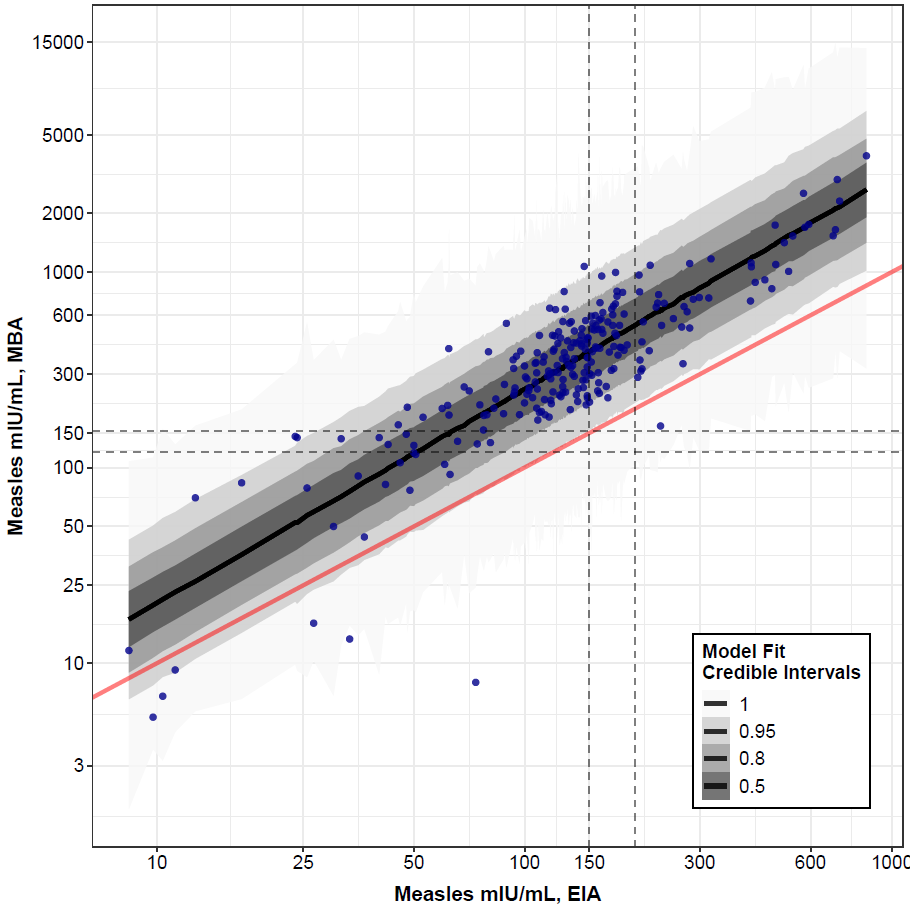


**Fig 5. Log-Log Model Fit (N=249).** The blue points represent the data. The black line indicates the mean fitted regression line, while the red line indicates a reference line of equivalence between the EIA and MBA. The ribbons of different shades of grey represent the 50%, 80%, 95%, and 100% credible intervals. The dashed line on the y-axis indicates the MBA cutoff for seroprotection of 153 mlU/mL and 120 mIU/mL. The dashed lines on the x-axis indicate the thresholds for borderline (150 mIU/mL) and positive (200 mlU/mL) in assessing immune status using the EIA.


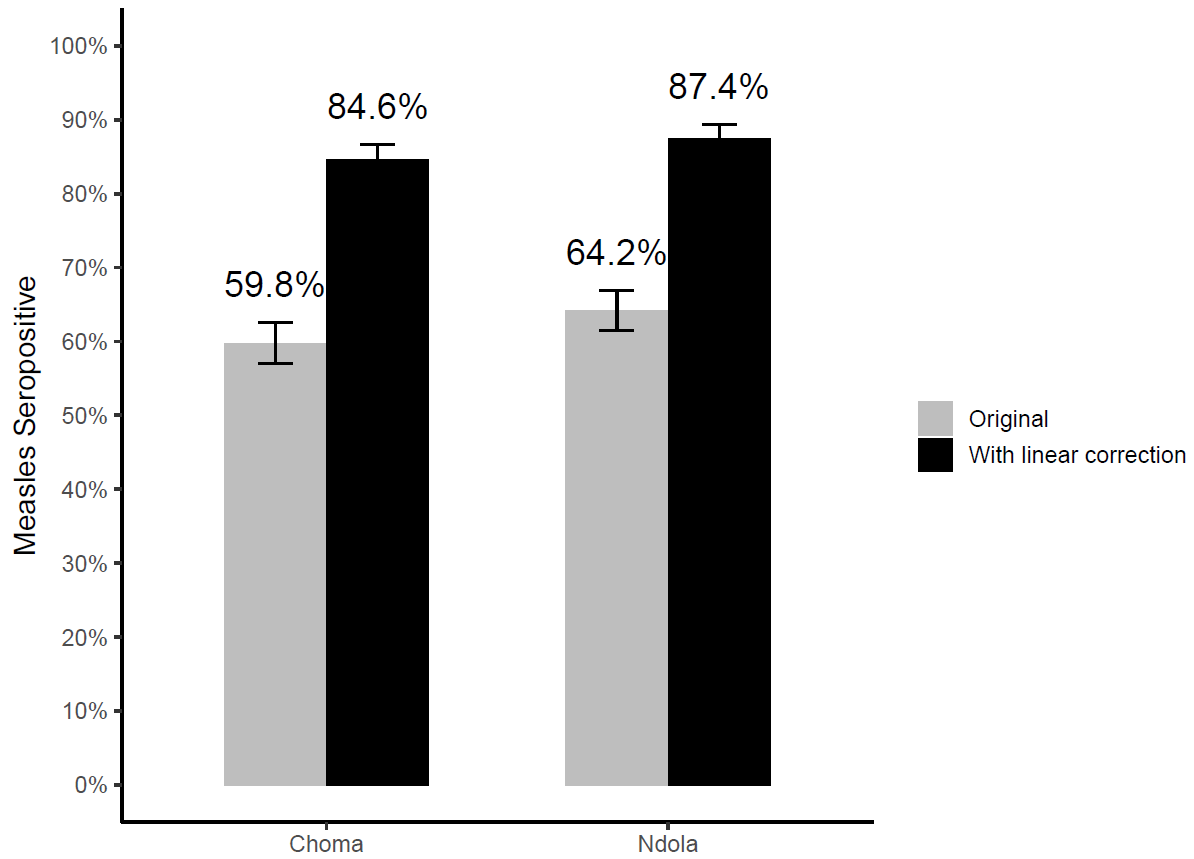


**Fig 6. Measles seropositivity before and after applying the correction factor.** The unadjusted results represent the IgG antibody concentrations obtained from the EIA; values above 150 mIU/mL were considered seropositive (i.e., borderline values of 150-199 mIU/mL were considered positive). The adjusted results represent the IgG antibody concentrations obtained from the EIA with the correction factor applied; values above 153 mIU/mL were considered seropositive.

**References**

1. Winter AK, Takahashi S, Carcelen AC, Hayford K, Mutale W, Mwansa FD, et al. An evaluation of the early impact of the COVID-19 pandemic on Zambia's routine immunization program. PLOS Glob Public Health. 2023;3:e0000554.

2. Carcelen AC, Winter AK, Moss WJ, Chilumba I, Mutale I, Chongwe G, et al. Leveraging a national biorepository in Zambia to assess measles and rubella immunity gaps across age and space. Sci Rep. 2022;12:10217.

3. Hayford K, Mutembo S, Carcelen A, Matakala HK, Munachoonga P, Winter A, et al. Measles and rubella serosurvey identifies rubella immunity gap in young adults of childbearing age in Zambia: The added value of nesting a serological survey within a post-campaign coverage evaluation survey. Vaccine. 2019;37:2387-93.

4. Murhekar MV, Gupta N, Hasan AZ, Kumar MS, Kumar VS, Prosperi C, et al. Evaluating the effect of measles and rubella mass vaccination campaigns on seroprevalence in India: a before-and-after cross-sectional household serosurvey in four districts, 2018-2020. Lancet Glob Health. 2022;10:e1655-e64.

5. Coughlin MM, Matson Z, Sowers SB, Priest JW, Smits GP, van der Klis FRM, et al. Development of a Measles and Rubella Multiplex Bead Serological Assay for Assessing Population Immunity. J Clin Microbiol. 2021;59.
