# Supplementary material for: High measles and rubella vaccine coverage and seroprevalence among Zambian children participating in a measles and rubella supplementary immunization activity
Source: PLOS Glob Public Health. 2025 Aug 29;5(8):e0003209. doi: 10.1371/journal.pgph.0003209 (PMC12396667; doi:10.1371/journal.pgph.0003209)
Supplement: Attachment — Submitted filename: Manuscript Number.docx [file pgph.0003209.s012.docx]

Manuscript Number: PGPH-D-24-00874

*High measles and rubella vaccine coverage and seroprevalence among Zambian*

*children participating in a measles and rubella supplementary immunization activity*

Remark

It would have been good if the authors had briefly written about the selection criteria of the study sites.
