# Supplementary material for: High measles and rubella vaccine coverage and seroprevalence among Zambian children participating in a measles and rubella supplementary immunization activity
Source: PLOS Glob Public Health. 2025 Aug 29;5(8):e0003209. doi: 10.1371/journal.pgph.0003209 (PMC12396667; doi:10.1371/journal.pgph.0003209)
Supplement: Attachment — Submitted filename: Review_response_17July2024.docx [file pgph.0003209.s013.docx]

Manuscript Review Response: PGPH-D-24-00874

High measles and rubella vaccine coverage and seroprevalence among Zambian children participating in a measles and rubella supplementary immunization activity

PLOS Global Public Health

Dear Dr. Flaxman and Reviewers of PGPH-D-24-00874,

We want to thank you all for thoughtfully reviewing our manuscript and providing your insight and assessments. We believe these comments were highly valuable and have guided us to provide a more robust and clearly written manuscript, which will aid in it being useful to the public health community and researchers. We have attempted to respond to all comments and have made edits to the submitted abstract for many of them. We have listed out the comments below and included our responses to them in red for easy re-review. Tracked changes versions of the manuscript and supplemental information have been included in the re-submission.

Sincerely,

Shaun Truelove
Johns Hopkins Bloomberg School of Public Health

**Reviewer #1:**

1. Abstract: The method section of the abstract discusses only data collection. I think it would be beneficial for readers to have a summary of the methods used. In the results section of the abstract, the authors provide an estimate for the percentage of children that wouldn’t have received MR dose 1 or MR dose 2. I almost always find it peculiar when people use the term "estimate" without showing the uncertainty levels. Of course, there is a time and place for everything, and this seems like the right one.

We have added a sentence noting these methods to the abstract, specifically “We used the expected vaccination probability by age derived from Demographic and Health Surveys to quantify the value of the immunization campaign, estimating the proportion of children who would not have otherwise received routine MR doses.”

Additionally, we originally kept uncertainty levels out of the abstract to meet word limits. We have added these back in now.

2. Introduction: Very well written and succinct. Just one comment: I believe paragraph two can benefit from more references.

Thank you! We have added a reference on line 77.

3. Methods: Survey setting- This paragraph is not entirely clear to me, “For facilities with multiple outreach sites, the survey teams moved with the vaccination team to the different locations where possible; otherwise, sites expected to have the most children during the campaign were selected.” Overall, how frequently were these decisions made?

We have modified this paragraph in an attempt to make this clearer. In general, the study staff was not involved in any decisions regarding movement of vaccination teams or selection of outreach sites - our goal was to simply capture a sample of the children being vaccinated within this campaign as it happened according to standard practice. The central study team, including researchers from Johns Hopkins and the research sites, decided on which outreach sites would be included based on the district SIA microplans and local context in terms of accessibility and population characteristics in the catchment area. Here is the modified text, starting on line 119 in the manuscript:

Revised text: “Five facilities in each district were selected to have serosurvey enrollment at both fixed and outreach posts, for a total of 30 campaign sites between the two districts. Outreach sites expected to have the most children during the campaign were selected. If the outreach site varied by day during the campaign for a given facility, the survey teams moved with the vaccination team to the different locations where possible. Decisions regarding selection of outreach sites and movement of survey teams were made by the central study team based on the campaign microplan.”

4. Methods: Statistical analysis: First of all, however simple or complex the model one is using, one must always include mathematical/statistical formulas. For one, it makes understanding easier and, most importantly, it reassures that the authors have fully understood the implications and the use of the model/method. Second, I am not exactly sure if the comparison outlined in this paragraph is a fair comparison: “Estimates of measles-rubella vaccine dose 1 (MR1) and dose 2 (MR2) vaccination status among children 12 to 23 months of age and 24 to 35 months of age, respectively, were compared with 2020 estimates from WHO-UNICEF (WUENIC).”

The comparison was specifically defined in order to be a direct comparison. WHO-UNICEF (WUENIC) estimates are defined for specific age groups, specifically 12-23 months of age for the first dose of MR and 24-35 months for the second dose of MR.

Third, the authors have indicated that the association between vaccination status and day of SIA and other child-level characteristics were explored with logistic regression. For me, the use of the term “other child-level characteristics” is a bit concerning. What are these “others”? If included, please point me to the right place in the paper.

We have added details to this sentence to detail the characteristics examined (line 171-173):

“Associations between vaccination status and day of the SIA and other child-level characteristics, including, including urban/rural, fixed/outreach vaccination site, travel time to the campaign, vaccination card availability, number of siblings below 5y, missing DTP1, and child’s age.”

Furthermore, the authors implied they used predicted probabilities for MR1 at 12 months or MR2 at 24 months. There are many ways to construct probabilities; it's not clear what has been used. Moreover, the statement, “Predicted probability of MR1 at 12 months, or MR2 at 24 months, was estimated for each of the 30 SIA sites using district-specific logistic regression models adjusted for age in years to account for differences in age distribution by SIA site,” seems very vague as far as models go. Clear specifications of the model(s) and rationale (I mean substantive ones), for choosing this model, are needed.

We do not believe adding these details to the main text is appropriate for this manuscript and will complicate and detract from it, thus we have included it in the supplement and added a reference to the supplement (see line 176). This text in the supplement is as follows:

We estimated district-specific predicted probability of a child 12 months of age having received MR1 or a child 24 months of age having received MR2 using a logistic regression model adjusted for age and campaign site. The model for MR2 was restricted to children 24 months and older.

We have added the following text detailing the logistic regression models to the supplemental information (see pp. 1).

“We conducted district-specific logistic regression models with MCV receipt (1 or 2 doses) as the binary outcome and campaign site as the predictor, adjusted for the child’s age. The model for MCV2 was restricted to children ≥ 2 years of age. Using the output from these models we estimated the predicted probability of a child 12 months of age having received MR1 or a child 24 months of age having received MR2 at each campaign site.

MCV1 receipt ~ SIA site + child’s age

MCV2 receipt ~ SIA site + child’s age [restricted to children ≥ 2 years]”

The same comment goes for the logistic regression model used to study association between measles seropositive and child-level characteristics. My argument is that with proper substantive justification, one can always achieve a higher level of rigor using more advanced model setup (not always, the simpler ones can be well justified as well).

We are not sure we follow or agree that a more advanced model setup always (or even most times) achieves a higher level of rigor. Instead, it is more important to define an appropriate model that captures the associations one is interested in, often without over-complicating the model. We have included the model description text in with that noted above in the Supplement.

“We conducted district-specific logistic regression models with MCV receipt (1 or 2 doses) or seropositivity as the binary outcome and the child-level characteristic as the primary predictor (urban/rural, fixed/outreach vaccination site, travel time to the campaign, vaccination card availability, number of siblings below 5y, or missing DTP1), adjusted for the child’s age. The model for MCV2 receipt was restricted to children ≥ 2 years of age.”

5. Methods: Effectiveness and efficiency of vaccination activities: In general, I remain a bit skeptical about how the effectiveness of vaccination activity was measured, especially whether the Odds Ratio (OR) comparing the SIA values with the general population is robust, given the many biases at play here (from both). A strong justification for the validity of this method might be required from the authors. The same thing also applies to how efficacy was estimated; in addition, for efficacy, the use of the 2018 Demographic and Health Survey and the 2020 SIA was not fully described (temporal variation). Moreover, personally, for the objective or measurement the authors are trying to make here, a more nomenclature, which is not so broad and general, and which is very specific, can be used.

Thanks for this comment - it’s clear we need to add some text to define this a bit better. We have modified the Methods text to include some additional details. We add the following on lines 226-229:

“A *VAEC* of 1 is the best possible value, indicating the vaccination activity reached a child who truly needed the vaccine with every dose given; in contrast a very low *VAEC* indicates an inefficient activity: *VAEC*=0.05 indicates it required 20 doses to reach 1 child who truly needed the vaccine.”

We also fixed an error where we used “vaccine activity efficacy” instead of “vaccine activity efficiency” (lines 195, 225).

We also have added additional details in the Supplemental Materials to fully explain these methods and their justification (pg 1-2).

We apologize, but we are not sure what you are asking by “Moreover, personally, for the objective or measurement the authors are trying to make here, a more nomenclature, which is not so broad and general, and which is very specific, can be used.” If you are suggesting more specific names for the metrics, while we agree, these are rather broad, we believe they capture the goals of the metric. But we have added acronyms for more specificity, VAET and VAEC. Hopefully this helps.

6. Results: For the results section, I will only have one question (when I have more clarity and more justification for choices of methods in the method section, I can get back to this); however, the 75% and 91% agreement for participation in the serosurvey study across the two districts is significant. I wonder if there are some theories behind the differences between these two?

Refusals for the serosurvey in Choma District were concentrated in three campaign sites, including two outreach sites, where we observed 39% or higher refusal. Two of the three sites in Choma District with higher refusals for the serosurvey were outreach sites which tend to serve harder to reach communities. The communities surrounding the three sites in Choma District have a high population of Zionists and Seventh Day Adventist Christians who are against blood collection. There were also concerns with the blood being used by Satanists for ritual purposes. This is consistent with a finding from a prior study also done in Southern Province (see below).

Carcelen AC, Limaye RJ, Mutembo S, Hamahuwa M, Thuma PE, Moss WJ, et al. Acceptability of serosurveys in southern Zambia: data collector and caregiver perspectives. Discov Soc Sci Health. 2023;3(1):3.

**Reviewer #2:**

1. It would have been good if the authors had briefly written about the selection criteria of the study sites, which would have given much more clarity in selecting the study sites. With this modification the manuscript may be accepted for publication.

Since this was a demonstration project to nest a serosurvey within an SIA we focused on two districts in different settings (rural and urban). There were also feasibility considerations, as these districts are where research partners are located. Information on how the campaign sites were selected is provided starting on line 109 under ‘Survey setting’.
