# Supplementary material for: High measles and rubella vaccine coverage and seroprevalence among Zambian children participating in a measles and rubella supplementary immunization activity
Source: PLOS Glob Public Health. 2025 Aug 29;5(8):e0003209. doi: 10.1371/journal.pgph.0003209 (PMC12396667; doi:10.1371/journal.pgph.0003209)
Supplement: Attachment — Submitted filename: Review_response_7Nov2024.docx [file pgph.0003209.s014.docx]

Manuscript Review Response: PGPH-D-24-00874R1

High measles and rubella vaccine coverage and seroprevalence among Zambian children participating in a measles and rubella supplementary immunization activity

PLOS Global Public Health

Dear Dr. Flaxman and Reviewers of PGPH-D-24-00874R1,

We want to thank you all for thoughtfully reviewing our manuscript and providing your insight and assessments on the revision we submitted. We believe these comments were highly valuable and have guided us to increase the clarity of the manuscript. We have attempted to respond to all comments and have made edits to the submitted abstract for many of them. We have listed out the comments below and included our responses to them in red for easy re-review. Tracked changes versions of the manuscript and supplemental information have been included in the re-submission.

Sincerely,

Shaun Truelove
Johns Hopkins Bloomberg School of Public Health

**Reviewer #1:**

A. Abstract: Clarity: “We used the expected vaccination probability by age derived from Demographic and Health Surveys to quantify the value of the immunization campaign.” Can the author use more descriptive words instead of “value”?

While we recognize the use of the word “value” is a somewhat vague term here, what we are describing is broad, and includes multiple interpretations of “value”, including the benefit the immunization campaign provides to reducing susceptibility at the time of the campaign, the eventual benefit to reducing susceptibility accounting for how many children would have been vaccinated eventually without it, and the efficiency of the campaign to do this (i.e., doses needed to reach susceptible children). The “value” of the campaign under these metrics depends on the audience and the goals. As such, without increasing the length of the abstract substantially, we elected to use a broader term that captures the full picture and believe this does not necessitate changing.

B. Introduction:
1. In paragraph 2, “...but their results are often subject to bias, substantial uncertainty, and...” What kind of bias or uncertainty are the authors implying? Be as clear and specific as possible. Given the objective of the paper, shouldn't the description of the gaps in post-campaign surveys focus on the fact that they cannot answer how many susceptible (as defined by the paper) children received the vaccine?

We recognize the lack of specificity and appreciate you identifying this. We have revised this paragraph to add specifics about the PCS limitations in lines 71-80:

“Little is known about the proportion of susceptible children vaccinated during MR SIAs. Traditionally, we rely on post-campaign coverage surveys (PCS), conducted shortly after an SIA, to estimate the number of zero-dose, previously unvaccinated children vaccinated during the SIA. However, due to the nature of these PCSs, the usefulness of these surveys to quantify the effectiveness and value of SIAs is limited: their results can be subject to biases in sampling and response, favoring individuals with greater accessibility and often those who would be more likely to receive vaccination, as well as substantial uncertainty as a result of relying on recall by care-givers to identify those who received SIA doses. Further, because they are conducted after the SIA, they cannot assess whether SIA-vaccinated children were truly seronegative to measles and rubella viruses [6]. Direct measurement of the quantity and proportion of SIA-vaccinated children who were susceptible prior to the SIA can demonstrate the added value of SIAs in closing population immunity gaps.

2. In the same paragraph, “Understanding whether susceptible...” could be improved by using more objective statements like “estimating” instead of “understanding.”

We have revised this to be more specific:

“Direct measurement of the quantity and proportion of SIA-vaccinated children who were susceptible prior to the SIA can demonstrate the added value of SIAs in closing population immunity gaps.”

3. “Zambia has a strong childhood vaccination program with 93% coverage in 2019 for the first dose of the measles-rubella (MR) vaccine but only 66% for the second dose.” Can this be phrased differently? The first part of the sentence and the last part seem conflicting.

We have revised this to be less contradictory:

“Zambia has a strong childhood vaccination program. They have consistently maintained high coverage of the first routine dose of measles-rubella (MR) vaccine, with 93% coverage in 2019. While second dose coverage is substantially lower at 66%, the Zambian Ministry of Health has made up for this with a long history of successful SIAs, including one during Child Health Week in November 2020 [12, 13].”

4. In the same paragraph, the authors indicated that the 3 million doses delivered during Child Health Week might reflect suboptimal coverage. How does this 3 million translate into coverage? Would it be suboptimal? If not, could COVID-19 and operational challenges explain the subsequent lack of protection for outbreaks?

We have rephrased this a bit to be clearer. And yes, while there were 3 million doses, this is estimated to be only 68.6% coverage (Yang et al, Vaccine, 2023). It was suboptimal, and the disruptions in coverage also likely led to lower vaccination. We have rephrased this section to be:

“Despite this fairly high vaccination coverage and a recent SIA, measles outbreaks were reported in several districts in 2022 and 2023, following the COVID-19 pandemic. While the drivers of these outbreaks is not fully known, it is speculated they may be the result of lower than optimal coverage of the November 2020 MR vaccination campaign, which is estimated to be 68.6%, and disruptions to the routine immunization program during 2020-2022 from the COVID-19 pandemic [14, 15].”

C. Methods:
1. The first paragraph of the method section may need a reference.

The district-level vaccination coverage reported in this paragraph is based on administrative coverage data from DHIS2. We have specified that this is unpublished administrative coverage data.

2. “These characteristics included geographic location and setting (urban versus rural), type of health facility, size of the health facility catchment area, functionality of the health facility based on national performance metrics, and details on accessibility of the health facility or difficult-to-reach subpopulations in the catchment area.” Were all these details acquired from site staff? If so, I am just thinking of the different limitations the authors can include in their discussion section.

These details were acquired from site staff and our experience working in this setting. We already have limitations statements that touch on this, and we added an additional sentence to include the limitation of the number of sites and districts:

“Further, as we only enrolled a relatively small sample of the children attending the campaign, it is also possible that the study children do not fully represent all children who attended the SIA in these districts. Additionally, with 30 sites from two of 116 districts, this study may not be fully representative of children in Zambia.”

3. If I missed it, I apologize, but is the travel time data from the participants (caregivers) or from site staff? In both cases there will be a limitation worth acknowledging, as objective measurement would require considering factors like different terrains, transportation availability, and other logistical elements.

Yes, travel time to vaccine sites by caregivers was collected and analyzed, see Table 1. Further, while these data are interesting, they do not necessarily add substantially to this analysis because we do not know anything about those children who did not attend the SIA, thus we did not include them prominently.

4. “Five facilities in each district were selected to have serosurvey enrollment at both fixed and outreach posts, for a total of 30 campaign sites between the two districts. Outreach sites expected to have the most children during the campaign were selected.” This statement is repetitive; the previous sentence already conveys the same information.

Thank you, this has been resolved by removing the prior sentence.

5. In the last paragraph of the Specimen collection and testing section, “A stratified random subsample of 300 DBS specimens was selected from those collected in Ndola District.” Why only Ndola District (just wanted to know)?

Logistics and sample size statistics. It was more efficient to have specimens collected and shipped from a single district, rather than attempting to ship from two districts. Additionally, selecting them from a single district enabled more robust statistical comparison between the ELISA and MBA.

6. In this same paragraph there is an abbreviation for EIA, which was never defined before.

Thank you, this has been fixed.

7. In the statistical analysis section, the authors specified they used the vaccination card as one variable in their logistic regression model for the outcome, vaccination status. Could you provide a brief description of the perceived association between these two and the need for controlling this especially related to other related variables that the authors already controlled for (just for my understanding, no need to change anything)?

The variable is vaccination card availability, in contrast with vaccination recall. We include this as vaccination card is expected to be much more reliable at predicting vaccination status than recall by a caregiver.

D. Results:
1. Table 1 doesn’t contain all the variables used in the model. Can it be made more comprehensive? (I am assuming this table was intended for the variables used in the main analysis. If not, and the choice of what was included is intentional, feel free to ignore this comment.)

Table 1 was meant to describe the children in the study and note critical variables as well as differences between districts, and not to describe the logistic regression. Variables included were intentional.

2. Maternal education, looking at secondary or higher, is highest in Ndola, which aligns with expectations given that Ndola is an urban district. However, for primary or less, Choma is higher, and I believe much of the percentage in this category comes from individuals with less than primary education, which I expect to be higher in Choma. If the second category were changed from “primary or less” to “primary” as one group and “less than primary” as a separate group, the percentage distribution might change and this might have significant impact on the significance level and estimated coefficient of the model.

Multi-level versions of the maternal education variable were explored, but did not demonstrate notable differences. We are not aiming to understand the minute details of the impact of maternal education in this analysis – it has been shown numerous times that education affects vaccination coverage, though this differs across populations. To limit the complexity and aid in interpretability, we believe leaving this variable as 2-level is appropriate.

3. In the Vaccination status section: “(n=2,364; 98.5%), 94% (n=2,214).” The author should ensure consistency in the writing format.

Thanks for noting this. We have revised this to be consistent in the noted section.

4. In paragraph 3 of the results section, the authors use “vaccination activity effectiveness.” They should consistently use the terminology they coined throughout the paper (VAET).

Good catch. Fixed.

5. In paragraph 3 of the results, the authors refer to “Lower maternal education.” Does this refer to primary or less education?

As seen in Table 1, this refers to secondary education or higher compared to primary or lower.

6. In the Measles and rubella seroprevalence section, the term “two sites” is used. The term "site" has been inconsistently applied throughout the paper—sometimes referring to hospitals (30 SIA sites) and now to districts. It would help to maintain consistency.

This has been revised to state “two districts” on lines 337-338. We have also revised the paragraph in lines 118-130 to clarify:

“In each district, we purposefully selected 10 MR SIA vaccination facilities per district to capture the range of characteristics. The selected facilities included a mix of rural health centers, urban health centers, hospital-affiliated health center, and health posts. Health facility- and community-level characteristics were obtained for each facility with input from the study staff. These characteristics included geographic location and setting (urban versus rural), type of health facility, size of the health facility catchment area, functionality of the health facility based on national performance metrics, and details on accessibility of the health facility or difficult to reach subpopulations in the catchment area. Five facilities in each district were selected to have serosurvey enrollment at both fixed sites and outreach posts, while the other five facilities only included enrollment at fixed vaccination sites, for a total of 30 campaign sites between the two districts.”

E. General Comment
1. For anyone who is skeptical of the methods used, how can the authors demonstrate beyond reasonable doubt that the methods throughout the paper are robust? This could involve considering sensitivity analyses, changing some assumptions, or re-evaluating how the unknown status was treated. Obviously, there are so many ways to show robustness.

This comment seems a little non-specific. Which methods are you referring to? We think you are referring to the novel metrics proposed in this manuscript. The use of these manuscripts is intended to introduce these metrics and not robustly prove the values they produce in this analysis. Further, they are generally susceptible to uncertainty in the data, but there are very few assumptions made with them. Because the robustness of these metrics is not defined by their calculation or assumptions, but rather by the uncertainty or bias in the data that go into them, we do not believe it was necessary to conduct sensitivity analysis. This is particularly the case because this manuscript is meant as an introduction to them, and these metrics are meant to be practical metrics that enable interpretable assessment and comparison across immunization activities. Any such comparison would need to make consistent assumptions across activities (i.e., treatment of unknown status).
