# Supplementary material for: High measles and rubella vaccine coverage and seroprevalence among Zambian children participating in a measles and rubella supplementary immunization activity
Source: PLOS Glob Public Health. 2025 Aug 29;5(8):e0003209. doi: 10.1371/journal.pgph.0003209 (PMC12396667; doi:10.1371/journal.pgph.0003209)
Supplement: Attachment — Submitted filename: Review_response_3Jul2025.docx [file pgph.0003209.s015.docx]

Manuscript Review Response: PGPH-D-24-00874R1

High measles and rubella vaccine coverage and seroprevalence among Zambian children participating in a measles and rubella supplementary immunization activity

PLOS Global Public Health

Dear Dr. Campbell and Reviewers of PGPH-D-24-00874R2,

We want to thank you all for thoughtfully reviewing our manuscript and providing your insight and assessments on the revision we submitted. We believe these comments were highly valuable and have guided us to increase the clarity of the manuscript. We have attempted to respond to all comments and have made edits to the submitted abstract for many of them. We have listed out the comments below and included our responses to them in red for easy re-review. Tracked changes versions of the manuscript and supplemental information have been included in the re-submission.

Sincerely,

Shaun Truelove
Johns Hopkins Bloomberg School of Public Health

**Reviewer #3:**

I have only minor optional suggestions for clarification.

1. The one I feel most strongly about is that the language about regression should clarify whether the regression accounted for the complex sample design of the study. Was it done with something like the ‘survey’ package in R or the svy: prefixes in Stata? If not, I think it should be re-done. All other comments are just suggestions.

Response: For this study we purposefully selected 10 vaccination facilities per district. Five facilities in each district were selected to have serosurvey enrollment at both fixed sites and outreach posts, while the other five facilities only included enrollment at fixed vaccination sites, for a total of 30 campaign sites between the two districts. Children aged 9 months to 5 years who were vaccinated at the selected campaign sites were systematically sampled for the serosurvey, with sampling designed to enroll a similar number of participants at each health facility and distribute enrollment across the days of the campaign. Although it was a multi-stage sampling design, we did not use a probabilistic sampling design for the selection of the health facilities and did not intend for the results to be generalizable to the district level or beyond the children attending the campaign at the selected health facilities. This is noted as limitation in the Discussion section (paragraph starting on line 426). Therefore, we did not use the survey package because this is an unweighted analysis.

2. The estimation of hazard, or the probability that each un/under-vx’d child would or would have not received the dose otherwise is an interesting element of this work. I would like to see in one of the Annexes some documentation of the sample size on which those models were based. How many children age 12-23 and 23-35 were in the DHS dataset that you used to estimate the hazards? It might be helpful to see a table listing number of children by months in age and the % of those that had received MCV1 and MCV2. But at the very least, please report the total N for each cohort and each district.

Response: Thank you for your request to provide more detail. The hazard is dependent on the district- and age-specific probabilities of vaccination. We have provided more detail in the supplement, including a reference to where the original estimates were generated (Winter et al PLOS Glob Pub Heal 2023). In summary, we used a conditional spatial autoregression spatial model to estimate both the rate of vaccination over age and the lifetime probability of vaccination for each district in Zambia. From this we generated the age-specific vaccination rate for Ndola and Choma districts, which contributed 144 and 132 observation of 5,670 total observation in Zambia across 112 districts.

3. Was there a post-campaign coverage survey for this SIA? If yes, please report the estimated campaign coverage from that work. If no, can you report an estimated coverage from administrative figures? It is one thing to say (line 89) that the campaign reached over 3M children…that sounds good…but for persons with an interest in measles SIAs, we want to judge the work against the benchmark of 95% target coverage. Please share what you know with the reader re: campaign coverage.

Response: Yes, there was a post-campaign coverage survey conducted for the SIA. The estimated campaign coverage (68.6%) and reference to a publication with the post-campaign coverage survey findings are mentioned in line 99. We have clarified in the text that this estimate is from the PCCS.

4. Line 127. The number 2.1 for DEFF is a curious value. Mention in a footnote, perhaps, how it was calculated.

Response: The DEFF was calculated based on the measles seroprevalence for a national seroprevalence serosurvey conducted in 2016 in Zambia as noted below. This was calculated using STATA 14.2 estat effects. We have added a reference to the manuscript.

Carcelen AC, Winter AK, Moss WJ, Chilumba I, Mutale I, Chongwe G, Monze M, Mulundu G, Nkamba H, Mwansa FD, Mulenga L, Rhoda DA, Hayford K, Mutembo S. Leveraging a national biorepository in Zambia to assess measles and rubella immunity gaps across age and space. Sci Rep. 2022 Jun 17;12(1):10217. doi: 10.1038/s41598-022-14493-3. PMID: 35715547; PMCID: PMC9204687.

5. Line 161. Clarify that your logistic regression accounted for the complex sample design. If it did *not* do so, then I strongly suggest that you need to redo it.

Response: Since we did not use a probabilistic sampling design for the selection of the health facilities and did not intend for the results to be generalizable to the district level or beyond the children attending the campaign at the selected health facilities, we did not account for complex sample design in the logistic regression.

6. Line 164. I applaud you for listing what kind of CI you calculated. Wald is not a good choice if the estimated prevalence approaches 0% or 100%. (See Dean & Pagano 2015 and many of the papers they cite.) I don’t require that you change it here, but for your future reference, I don’t think there is ever a good reason to ever use a Wald interval for a proportion. The better intervals like logit or Wilson or others approximate the Wald when the sample size is high or the outcome is near 50% and they only get better as the estimate goes to the extremes. Some options, like Clopper-Pearson are too conservative for usual use, but you’ll never go wrong using a Wilson interval which is also defined when the coverage is 0% or 100% (which some, like logit, are not.)

   Dean, Natalie, and Marcello Pagano. 2015. “Evaluating Confidence Interval Methods for Binomial Proportions in Clustered Surveys.” Journal of Survey Statistics and Methodology 3 (4): 484–503. <https://doi.org/10/gnjws5>.

Response: Thank you for flagging this oversight. We have updated the confidence intervals for seroprevalence to Wilson.

7. Effectiveness and efficiency: People have a difficult time thinking about odds ratios, so I’m not optimistic that these proposed measures will be widely used, but you have explained them clearly enough to use here.

Response: Thank you for the feedback. We plan to prepare a manuscript focused on this methodology using data from the 2024 post-campaign coverage survey following the September 2024 measles-rubella SIA in Zambia to increase interest and use of these metrics. We are also working with other countries on their post-campaign coverage surveys, so will hopefully replicate these metrics across countries as well.

8. Line 257. A point estimate of 39% probably shouldn’t have a 95% CI of 34-40%. Check the numbers. You said you were using symmetric Wald intervals. And even the asymmetric intervals all have the “long” tail pointing toward 50%...not pointing away from 50%.

We say we are using Wald (now changed to Wilson) intervals for seroprevalence estimates, not for the hazard and probability estimates. These estimates and their confidence intervals reflect the distribution of children’s ages in the study population. This population has right-skewed age distributions, meaning the lower bound has significantly more uncertainty, particularly for the probability of receiving a second dose, whereas the upper bound is fairly constrained by the data. See the distribution below. The reported confidence interval is correct so much as it’s driven by the data from DHS and the study population.

9. General comment: I like that many of your proportions omit the decimal place and any digits afterward. I think that improves readability. I find it districting when you switch back and forth between showing the decimal digit and not, as in the paragraph from lines 255-268.

Response: Thank you for the feedback. We have revised this throughout the manuscript.

10. Under limitations, mention that purposeful site selection is another reason that the sample may not be representative of all SIA participants.

Response: This point was clarified in line 436.

11. Under limitations, mention that variance calculations and confidence intervals in things like the regression analysis assume that the data are a representative sample; because this design used purposeful selection of data collection sites, we might think of the associations and outcomes here as being indicative but not representative.

Response: We have added a sentence in the Limitations paragraph (starting on line 432).

12. I recommend adding one or two more sentences about the children with no history of immunization whose serology indicated immunity to measles. These might be considered “previously zero-dose” children who did not need, in some sense, to be vaccinated as they were already protected. Is that presumably because they had contracted the disease when younger? This category of children is not commonly discussed in literature assessing the benefits of SIAs. Would there have already been an estimate of the % of zero-dose children who were immune to measles in Zambia? The serology is what could make a precise estimate of the prevalence known if the sample were random. Without serology, I suppose a PCCS could ask the caregiver if the child ever experienced measles. I know some PCCSs have asked that in Ethiopia, for instance, but of course many caregivers will not be able to report that detail of their child’s medical history accurately. I think those children warrant a few more sentences to describe how that outcome comes about and to say that perhaps they might not have been otherwise vaccinated, and so counted among the successes of the campaign, but they didn’t really need to be vaccinated, either.

Response: Of the 57 children with no history of immunization who were seropositive for either measles or rubella, 49/57 (86%) were seropositive for both measles and rubella. Given the low wild-type rubella circulation in Zambia, these findings suggest their vaccination status was misclassified and the child had previously received the measles-rubella vaccine through the routine system. Most of the zero-dose children prior to the campaign had a routine immunization card available (141/150) and measles seropositivity findings among the zero-dose children were similar when stratified by card availability (S5 Fig). This is described in the Results section in the paragraph starting on line 346. We have also added a sentence to line 392 in the Discussion.
